# The Embodied Self in Parkinson's Disease: Feasibility of a Single Tango Intervention for Assessing Changes in Psychological Health Outcomes and Aesthetic Experience

**DOI:** 10.3389/fnins.2016.00287

**Published:** 2016-07-07

**Authors:** Sabine C. Koch, Katja Mergheim, Judith Raeke, Clarissa B. Machado, Eliane Riegner, Joachim Nolden, Gudrun Diermayr, Dorothee von Moreau, Thomas K. Hillecke

**Affiliations:** ^1^Faculty of Therapeutic Sciences, SRH University HeidelbergHeidelberg, Germany; ^2^Department for Creative Arts Therapies and Therapy Sciences, Alanus UniversityAlfter, Germany

**Keywords:** embodiment, Parkinson's disease, Argentine Tango, dance movement therapy, arts therapies, body self-efficacy, beauty, aesthetic experience

## Abstract

**Background:** Dance is an embodied activity with benefits for mobility, balance, and quality of life (QoL) of persons affected by Parkinson's Disease (PD). It is enjoyable and likely to support adherence to movement prescriptions. The objective of this study was to assess the feasibility of measuring changes in psychological outcomes, specifically well-being, body self-efficacy, outcome expectations, and experienced beauty after a single Argentine Tango intervention in a workshop format. To anchor experienced beauty in a theory, the article introduces a *model of embodied aesthetics* featuring active art-making as a central aspect of healing in arts-based interventions.

**Methods:** In a single-group pre–post design, we tested the feasibility of measuring psychological changes of 34 PD patients from Southern Germany after an introductory workshop in Argentine Tango. They participated in a 90 min *Tango for PD* intervention and completed the *Heidelberg State Inventory* (HSI-24; (Koch et al., 2007)), the *Body Self-Efficacy* Scale (BSE; (Fuchs and Koch, 2014)) with a sub-dimension on *aesthetic experience*, and the *Credibility-Expectancy Questionnaire* (CEQ; (Devilly and Borkovec, 2000)) before and after the intervention. A subgroup completed the *therapeutic factors of arts therapies*-scale, a new measure to elaborate on the aesthetic experience. We analyzed pre–post-differences with a *t*-test for paired samples.

**Results and Discussion:** The study supports the feasibility of measuring health-related psychological changes from a single Argentine Tango intervention for PD patients, as well as acceptance and appropriateness of the intervention for the patient group. After the tango intervention, well-being, body self-efficacy, and outcome expectancies increased. Participants also experienced an increase in beauty of their movements and other aesthetic aspects. We suspect that, in addition to the functional and psychological factors identified so far, the aesthetic experience in dance may be an important therapeutic factor mediating several outcomes of dance and other arts-based interventions. A controlled study for evidence-based testing of targeted variables can now follow to examine the new hypotheses.

## Introduction

Parkinson's Disease (PD) is a progressive neurodegenerative disorder associated with movement disorders including impaired functional mobility and postural instability (Hackney and Earhart, 2010). The death of dopamine cells in the substantia nigra causes bradykinesia (slow movements), akinesia (absence of or impoverished voluntary muscle movements), resting tremor, rigidity (“stiff” movement, decrease in flexibility), freezing (no movement), and postural instability (Lindenbach and Bishop, 2013). Classical therapies for PD employing dopamine-receptor agonists or deep brain stimulation (DBS) improve PD symptoms but may cause side-effects such as drug-induced dyskinesia and surgical complications (Lindenbach and Bishop, 2013).

In recent years, there has been an increasing interest in non-pharmacological therapies and the effects of movement in general, and dance in particular, for people with PD. Integration of dance, and particularly tango, as an innovative approach into PD rehabilitation is supported by a number of recent reviews (e.g., Hackney and Bennett, 2014; Lötzke et al., 2015; Sumec et al., 2015; Abbruzzese et al., 2016) reporting dance-related effects on physical functions, as well as on cognitive and psychological outcomes such as depression level, enjoyment, and well-being. Here, we focus on psychological effects of *Tango for PD* from an embodiment perspective, and the aesthetic component that has not yet been taken into account as a mechanism. This component is therefore in this work first anchored in a theory model.

*An embodiment perspective* addresses the “living body” (“to *be* a body”—as opposed to the “body proper,” i.e., “to *have* a body;” Merleau-Ponty, 1962), that is the body–mind unity as an entity, and provides a timely framework to understand and explain phenomena we meet in Parkinson's Disease (Fuchs and Koch, 2014; Schiavio and Altenmüller, 2015, this volume). In our study, we drew upon embodied enactive approaches from cognitive and neurosciences (e.g., Niedenthal et al., 2005; DeJaegher and DiPaolo, 2007), clinical psychiatry and clinical psychology (Fuchs and Schlimme, 2009; Koch, 2011; Ramsayer and Tschacher, 2011; Michalak et al., 2015), and the arts therapies (Koch and Fischman, 2011; Koch and Fuchs, 2011; Schiavio and Altenmüller, 2015); we focused on the effects of a body-based intervention on body-based psychological outcomes. *Embodied perspectives* represent a novelty in understanding psychotherapeutic interventions and assume that our body is a therapeutic entry point that can operate relatively independently from the verbal level (Ramsayer and Tschacher, 2011; Michalak et al., 2015), but equally related to meaning. The body thus often provides an unexplored potential, a second option next to a verbal entry point to therapy. Embodied approaches assume that emotions are closely connected to our bodies (e.g., via embodied simulations; Gallese, 2001; Niedenthal et al., 2005), and that cognition and abstract concepts are based on sensorimotor processes (Lakoff and Johnson, 1999). The *enactive view* (Varela et al., 1991) draws from theoretical biology and looks at individuals as living systems. Principles of living systems are agency, plasticity (moment-to-moment adaptations), striving for balance, self-organization (Haken, 2010), autonomy, sense-making (DeJaegher and DiPaolo, 2007), embodiment, emergence, experience, and action-perception coupling as well as organism–environment coupling (Kelso, 1995). Enactive approaches have many of the same premises than embodied and dynamic systems approaches.

Embodiment approaches provide a framework from which PD and its effects are understood as a disorder of the *embodied self*, a concept focusing on the organismic and animated nature of human beings (Sheets-Johnstone, 1999; Fuchs and Schlimme, 2009), and the body–mind unity. The subjective bodily experience of the patient is taken seriously and the embodied interaction with the patient as experienced by the clinician is an important source of information. In *Tango for Parkinson*, movement and embodied therapies are employed in the service of increasing quality of life (QoL) and well-being. The body is the place where the illness happens, which endows it with an important role as a therapeutic entry point for increasing well-being and QoL. Bodily arousal and regulation mechanisms effect affect, attitudes, and behavior in coping with the illness. Our own previous work includes an embodied enactive theory framework for dance therapy (Koch and Fischman, 2011), a framework on embodied affectivity (Fuchs and Koch, 2014), and evidence-based studies on the effects of dance movement therapy on health-related psychological outcomes (e.g., Koch et al., 2014).

*Dance* has previously been investigated intensively in the older adult population showing that this relatively moderate form of physical activity yields improvements in balance and cognition (Kattenstroth et al., 2013; for reviews see Hwang and Braun, 2015; McNeely et al., 2015). While Lötzke et al. (2015) and McNeely et al. (2015) point out that there are still open questions in dance for PD, recent findings encourage its implementation into clinical practice. Studies report general positive effects of partnered dancing (Ashburn et al., 2014), music-based movement therapy (DeDreu et al., 2012), and social dance sessions (Lewis et al., 2016) on PD, with tango representing the most investigated form of dance for PD (Hackney et al., 2007; Sumec et al., 2015). Hackney and Earhart (2010) found that a 10-week program of dance classes improved balance, walking velocity and cadence among people with mild or moderate PD. This was true for non-partnered dance as well as partnered dance, whereas increased enjoyment and interest to continue the program was higher in the partnered dance group. In a similar vein, McKee and Hackney (2013) showed that community-based Tango lessons over 12 weeks improved spatial cognition, balance, and executive functions, while disease severity decreased, compared to a control-group receiving educational lessons. Duncan and Earhart (2012) found decreased PD severity and better physical functioning in a randomized trial comparing a tango intervention group with a control group. Safety issues of such tango interventions for PD patients have recently been addressed by Blandy et al. (2015; this volume).

In addition to effects on gait and balance (e.g., Fisher et al., 2008; Goodwin et al., 2008, 2011; Dibble et al., 2009; Morris et al., 2010; Duncan and Earhart, 2012, 2014), tango increased QoL (e.g., Hackney and Earhart, 2009), personal and social activities (Foster et al., 2013), and cognitive and psychological variables (Hashimoto et al., 2015). Support for the latter findings comes from a survey on the benefits of dancing among adults from Quiroga Murcia et al. (2009, 2010); participants reported that dancing affects emotional and physical aspects of health as well as social and spiritual domains and in particular self-esteem and coping strategies (Quiroga Murcia et al., 2010; see also Kreutz and Quiroga Murcia, 2015). In sum, however, meta-analyses and systematic reviews (e.g., Hackney and Bennett, 2014; Mandelbaum and Lo, 2014; Shanahan et al., 2014; Sharp and Hewitt, 2014; Lötzke et al., 2015) agree that the largest effects of *Tango for PD* exist for improving gait, balance, and QoL.

*Why tango?* Hackney and Earhart (2009) have found that Tango Argentino, with its improvisational nature, works better in improving gait, balance, and QoL in PD than ballroom dancing. One reason may be that patients are so focused on following and *attuning to the partner* in the present moment that they are not even realizing that they walk backward over extended periods of time, that they turn securely backward and forward, and that they repeatedly initiate movement without any problems. There have been recent findings on the *health improving nature of non-goal-oriented improvisational dance movement* that argue along the same lines (Wiedenhofer et al., 2016; Wiedenhofer and Koch, under review).

*How short can the intervention be* to still realistically lead to psychological effects? When Earhart (2009) or Duncan and Earhart (2012) talk about short-term interventions, they think of 6- to 10-week programs of Tango for PD (vs. 6- to 12-month programs that they have also been running). In our case, we assessed an ultra-short-term intervention in form of a 1.5 h workshop regarding feasibility, acceptance, and changes on psychological outcomes.

In conclusion, the *Tango for Parkinson* studies highlight the improvements in motor function for people with PD and high attrition rates in the dance groups compared to traditional exercise groups. While different motor symptoms were investigated by most of these studies, psychological variables are not well-understood. Duncan and Earhart (2012) stress the importance of additional work to explore the effects of exercises and dance on non-motor symptoms and activities of daily living. Therefore, the purpose of the exploratory part of the study was to assess the effects of a single intensive tango intervention on well-being, body-self efficacy, and patients' outcome expectancies.

In clinical practice, dance interventions can support adherence to keep high levels of daily movement and social activities, among other factors by causing pleasurable and aesthetic experiences from and with one's own body. The goal of this study was to employ tango for PD patients to explore its impact on health-related psychological outcomes in the course of assessing the feasibility of a workshop format, and to explore the aesthetic experience as a therapeutic factor, an aspect previously unaddressed. We anchor this aspect in the theory model of embodied aesthetics (Koch, 2016). This model can help us understand how dance therapy works from an arts therapies perspective.

**Figure 1:**
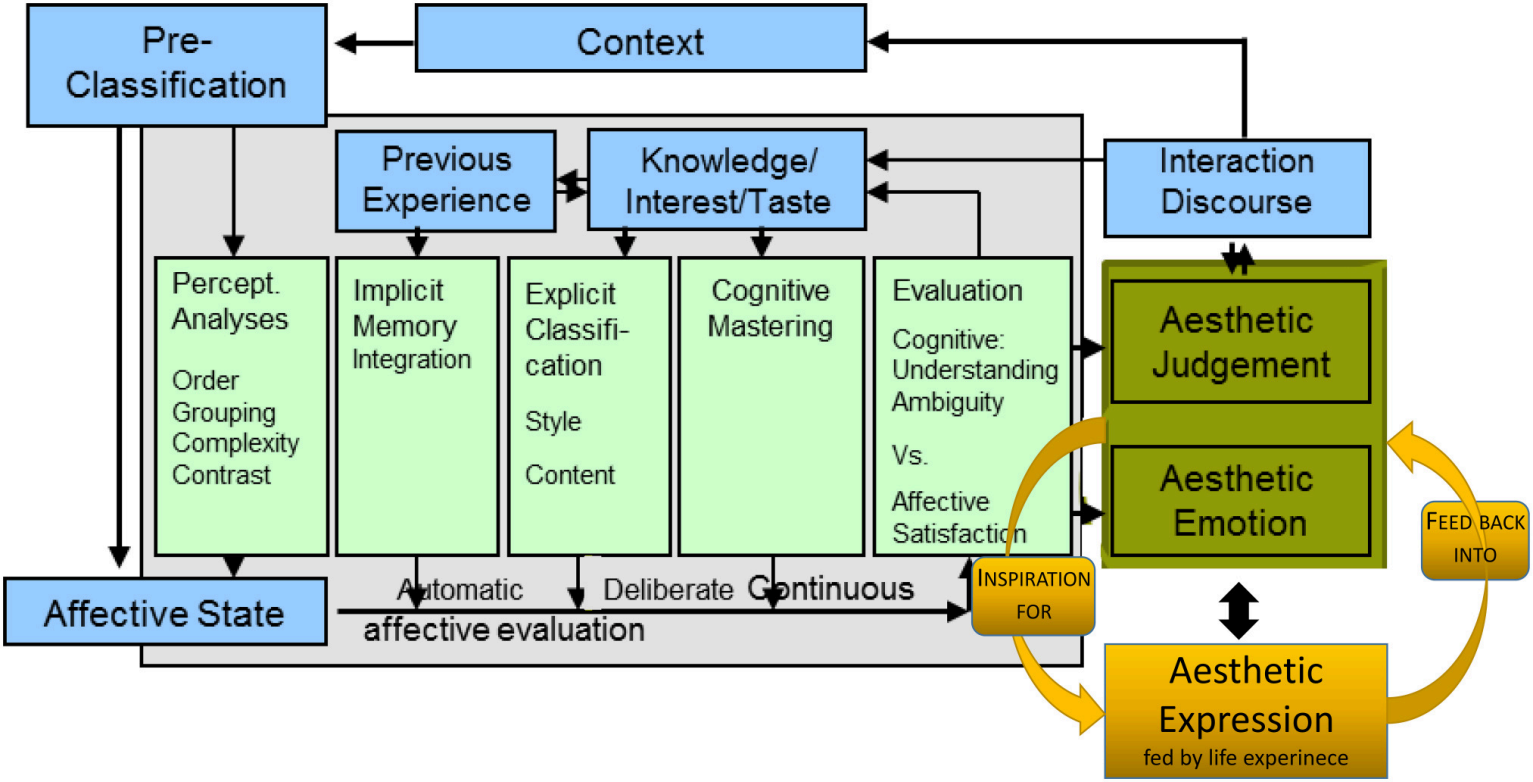
**Cognitive science model of aesthetic perception (Leder et al., 2004), extended by aesthetic expression ((Koch, 2016); new parts right hand side in orange)**.

Aesthetics has been defined as “a sensory experienced knowledge” by Baumgarten (1750/2007). Allesch (2006) pointed out that there is a lack of an aesthetic theory model suited for the arts therapies, and demands us to think big, that is to expand our thinking to include all forms of art, and not exclude nature, and everyday aesthetic phenomena. Given the lack of a theory model suited to explain the therapeutic factors of art-making, the model of embodied aesthetics was recently developed by Koch (2016) on the basis of the embodied affectivity model (Fuchs and Koch, 2014) and applies a circular causality and dynamic systems logic rather than a linear-causality logic (Thelen and Smith, 1994; Salvatore et al., 2015). The model extends the present state-of-the-art cognitive sciences model by Leder et al. (2004; Figure 1). As a classical input–output model, Leder's model focuses on the stages of cognitive and affective processing of an aesthetic stimulus with the result of a cognitive and an affective outcome (i.e., aesthetic judgment and aesthetic affect).

**Figure 2:**
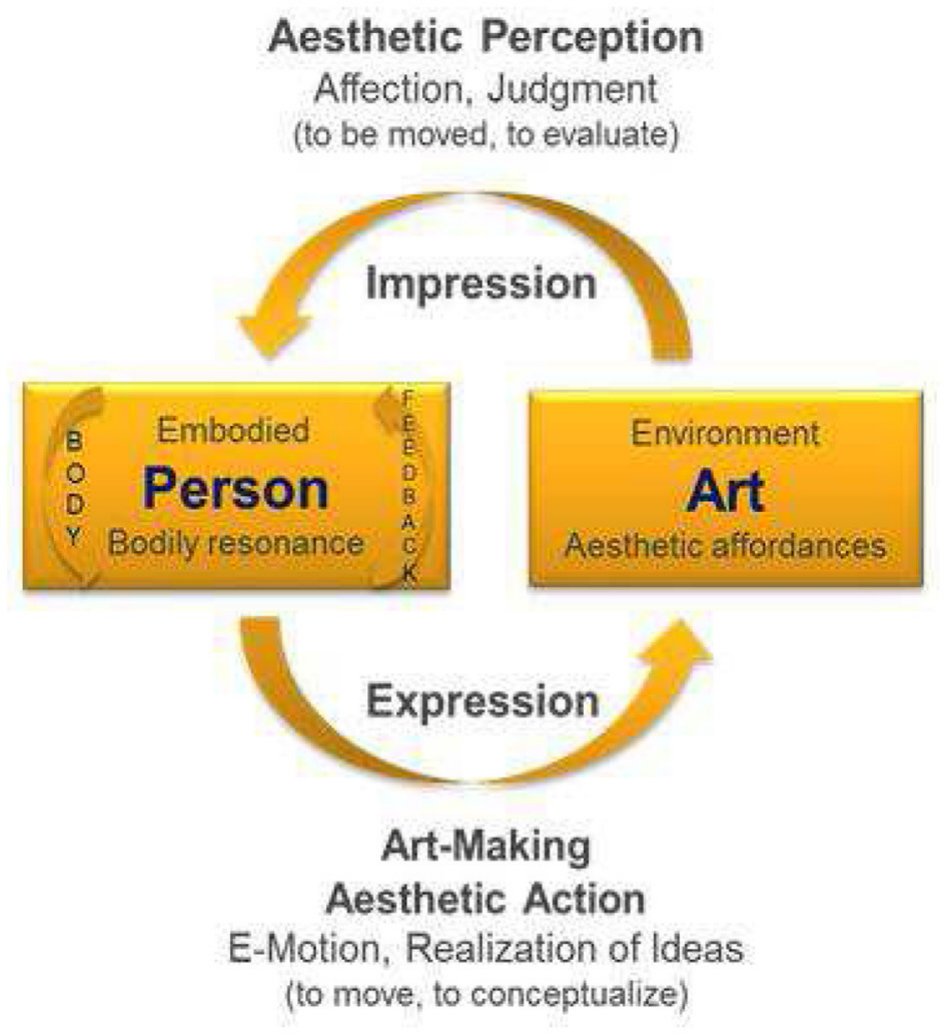
**Model of Embodied Aesthetics (Koch, 2016) including the perception and action side of aesthetics**. Since action (movement) is the basis of perception, but perception is also the basis for action (movement), we best talk about the unity of action and perception (cf. von Weizsäcker, 1940).

While the model of Leder et al. (2004) is a classical input–output model, the model of embodied aesthetics of (Figure 2) can be viewed both as an extended input–output model, and as a circular, systemic model, since input and output are in fact assumed to be parallel and not sequential processes. Such a “hybrid model” (of systemic and input–output model) at this stage allows us to investigate contexts with both experimental and dynamic systems methods. This is useful for our research context, as the evidence-based experimental studies in the health sciences are still regarded as the main pathway to advancing the general base of knowledge (whereas growing lines of science proceed to use systems models to be able to more adequately reflect the complexities of the “reality” of living organisms in living environments).

The two models are not exclusive of each other. The main point we want to make here is that the model of Leder et al. (2004) focuses solely on aesthetic *perception* (just as other cognitive science models of aesthetic experience; e.g., Martindale, 1984; Ramachandran and Hirstein, 1999; Reber et al., 2004; or updates of Leder's model by Chatterjee and Vartanian, 2014, or Leder and Nadal, 2014; even the model of Gifford, 1997, who bases his ecological aesthetic theory on Brunswick's lens model, is not helpful in terms of an embodied *enactive* perspective). Aesthetic action is missing. Yet, aesthetic action is central to the idea of healing and to the therapeutic process in the arts therapies.

The circular model of Koch (2016) complements aesthetic perception (impression side), addressed by the model of Leder et al. (2004; and other cognitive science models), with the previously unaddressed side of active art-making (expression) as practiced in creative arts therapies (Wallbott, 1982; Scherer and Wallbott, 1985; Wallbott, 1990; Fuchs and Koch, 2014; Koch, 2016). An aesthetic experience does not only result from an impression (perception) of art, but also from the expression of it (action). This experience may pass from mere playful expression and enjoyable experimenting (e.g., with music and dance), via a self-efficacy experience, for example, when moving or playing an instrument, to symbolic expression (e.g., how would your joy sound?), or the creation or formation of something beautiful/authentic in any arts modality.

The cycle is here described in movement, since movement provides the most immediate body feedback (Koch, 2014). The moment I move and experience my movement (e.g., as beautiful, authentic, or creative), I am affecting myself in a twofold way (cf. Merleau-Ponty, 1962, 1964): I am moving and being moved both at once—there is an overlay in sensory and motor-mapping and it is a mere attention issue where consciousness is focused on in a given moment; there is no movement without perception and no perception without movement (vgl. von Weizsäcker, 1940; Merleau-Ponty, 1964; Gibson, 1966). Likewise, when I stand in a museum absorbing a piece of art, my body resonates with it (my breath may reflects a change of my organismic system' arousal, my emotions may get involved, cognitive conflicts may emerge and strive for solution); when I see a movie, a theater piece, a music performance, my body resonates with it; my body is going into synchrony or asynchrony with it. This resonance is providing body feedback (aesthetic impression) and is also a starting point for aesthetic expression^1^.

In tango dancing there are external stimuli that initiate an organism–environment coupling in relation to the music, the partner, and the group. The “art product” is just a transient movement, a fleeting moment of beauty (or heightened authenticity). Music and partner affect and move the person in particular yet unpredictable and emergent ways that resonate within the lived body. The person's bodily resonance (kinesthetic) and the dance (kinetic) change and influence one another on a moment-to-moment basis (Merleau-Ponty, 1962, 1964; Koch, 2011), and bring body and mind into an experienced unity (Mainka, 2015; this volume). This may be observed, for example, by the synchronization of body rhythms on an individual as well as an interpersonal level (Koch, 2014; Edelhäuser et al., 2015; Heusser, 2015).

### The present study

The aim of our study was to show the feasibility of measuring health-related psychological changes following a single tango intervention, to ensure patient acceptance and that the workshop format is appropriate for patients with severe neurodegenerative health problems such as PD. We explored the following question (with the rationale for the selected outcome variables provided below): *Can a single tango-intervention improve well-being, outcome expectations, and body-self efficacy, including aesthetic experiences, in PD patients?*

(a) Well-being. In the course of PD, 54% of elderly patients show apathy, which in turn affects their QoL (Skorvanek et al., 2013). Depression, another non-motor symptom associated with PD, is prevalent in 68.1% of the PD patients (Chagas et al., 2013). Thus, the use of dance in order to stabilize affect, increase well-being and reduce depression (Koch et al., 2007) could be a useful approach for PD patients. We therefore explored whether a single tango intervention can increase well-being in PD patients.
(b) Body self-efficacy. Self-efficacy, the belief in one's abilities, is a construct, which is highly relevant for one's health-related coping mechanisms and actions (Schwarzer and Warner, 2013). Body self-efficacy refers to the body-related part of the skills (Fuchs and Koch, 2014). Since PD has many effects on the body level, bodily skills experienced as resources are an important factor to strengthen the resilience of PD patients. The idea that tango can increase patients' body self-efficacy has not yet been investigated in PD patients.
(c) Patient's therapy outcome expectancies. Patient expectancies of the therapy outcome play an important role in traditional psychotherapy research and can be an important predictor of the actual psychotherapy outcome (Johansson et al., 2011). Ametrano (2011) found that early outcome expectancies at the beginning of therapy significantly predict patient rated alliance. To our knowledge there is only one study in the field of dance movement therapy which explores the role of outcome expectancies with oncology patients (Mannheim and Weis, 2006), but none with PD patients. We wanted to test whether patient's outcome expectancies will increase after the tango intervention (Hypotheses 3). If this is the case then one might assume a higher alliance and attrition in the course of long-term interventions (Ametrano, 2011).
(d) Aesthetic experience. Aesthetic experience / experienced beauty was introduced as a secondary outcome in the course of the investigation when it became clear from the work in our continuously running *Tango for Parkinson* group at the SRH outpatient center that there were factors other than functional ones and the previously investigated psychological ones that supported patient attendance in the groups.

We assumed that well-being, body self-efficacy and outcome expectations increases after a single tango-intervention. Moreover, we expected an increase in aesthetic experience, particularly experienced beauty.

## Methods

### Sample

Thirty-four participants with PD from Southern Germany participated in three groups. Twenty-six participants were women and eight were men, the age range was 40–82 years (*M* = 60.5; *SD* = 11.06), with the mode at 50 years (*n* = 10), and thus a skewness on the younger side. Participants were recruited through contacts to the PD-support groups of Heidelberg, Sulzbach (Taunus), Mannheim, and Ludwigshafen. Two of them had had previously danced tango, 13 more reported to have formerly danced as a hobby. The live music was a new element for them. All but one patient successfully participated in the classes: the one patient in the second Heidelberg group sat from beginning to end and was not included in the data analysis. Most participants were pensioners (the regular retirement age in Germany is 65); their degree of handicap was between 50 and 100% with a mean of 72.78 and an *SD* of 18.31, nine persons were classified with a 70% handicap (mode), 5 with 50%, 5 with 100%, the remaining in between with a range from 40 to 100%. All participants were Caucasian. The study was carried out with written informed consent from all subjects in accordance with the Declaration of Helsinki, and followed the data protection requirements of the outpatient center at SRH University Heidelberg, without being separately submitted to an ethics committee.

### Procedure

The study was conducted in three introductory workshops to tango therapy for PD patients. Workshops took place at two different sites with two different dance movement therapists as session leaders. For an overview of the sample and settings, see Table 1. The first workshop was organized in February 2014 via the Sulzbach PD-support group who traveled to SRH University Heidelberg in order to participate in the study (18 participants, 7 partners). The second workshop was announced via the Heidelberg PD-support group and took place in June 2014 at SRH University of Heidelberg (5 participants, 1 partner), and the third workshop took place in May 2015 in the Ludwigshafen PD-support group setting (11 participants, 3 partners). In all three workshops participants first heard a presentation of ~30 min by the first author, head of the dance movement therapy Master Program at the SRH University Heidelberg, introducing them to the effects of arts therapies, particularly dance, movement, and music, on PD. Then participants provided informed consent and filled in the pre-test questionnaires (15 min). Then the tango intervention took place. After a short warm-up and introduction of the therapist (Workshop 1: Clarissa Barcellos Machado, dance movement therapist and tango teacher from Argentina; Workshop 2 and 3: Eliane Riegner, dance movement therapy advanced student and tango teacher from Germany), the participants were invited to join different exercises, for example, walking next to each other in pairs, one person leading, while the other person was following (e.g., walking backwards). They practiced basic steps of Tango Argentino first on their own and then in pairs. During the partnered dance, the PD patients worked with their spouses, relatives/friends, or students of the SRH Dance Movement Therapy Master Program as partners, or in rare cases with other patients. The entire intervention lasted ~90 min (for description of the intervention see Appendix A in Supplementary Material). The Argentine Tango instructor and dance movement therapist (CM) instructed in English and a German dance movement therapy student translated after each few sentences; this first workshop used recorded music, but the last three songs were accompanied by live bandoneon music. The second and third workshop, led by a tango instructor, and advanced dance and movement therapy student from Germany (ER), employed only recorded music. Participants in all three workshops were told that they could take their shoes off, if that was more comfortable for them, and that they could sit down and take a rest during the session whenever needed, which they did selectively. They completed the post-test immediately after the intervention (~15 min). Some needed help reading the items, which was provided by student helpers, however, all participants filled in the questionnaires by themselves.

**Table 1:** **Sample characteristics**.

	**Heidelberg 1 (2/2014)**	**Heidelberg 2 (6/2014)**	**Ludwigshafen (5/2015)**
*N* of PD patients	18	5	11
*N* of partners attending	7	1	3
Sex	13 w/5 m	4 w/1 m	9 w/2 m
Age	55.7	59.0	69.0
Nationality	17 German/1 Austrian	5 German	10 German/1 GB
Therapist	CM	ER	ER
Well-being	x	x	X
Body self-efficacy	x	x	X
*Two aesthetic items*	x	x	x
Expectancy outcomes	x	x	x
*Therapeutic factors scale*	–	–	x

*Sample characteristics, therapists, and scales employed; PD, Parkinson's Disease; ATs, Arts Therapies*.

### Materials and instruments

#### Material for the intervention

To find the correct body posture for Argentine Tango, the instructors used yellow “post-its” (sticky paper) that each patient attached to his/her sternum. The “post-it” symbolized a light that shines from the chest to the outside just like a beam. This device helped participants to find the correct upright posture, position of the spinal column, muscular tension, and convexity of the upper body for the dance and to connect to the partners via this point of the body.

#### Psychometric instruments (see Appendix B in supplementary material)

##### Psychological well-being

Well-being was measured by the 24-item “Heidelberg State Inventory” (HSI-24; Koch et al., 2007; unipolar version in the Appendix of Supplementary Material) with a range from “1” (“does not apply at all”) to “6” (“applies exactly”) assessing tension, anxiety, coping, positive affect, depressed affect, and vitality. The scale is based on a review of central outcomes of dance movement therapy by Goodill (2006). The internal consistency of the entire scale in previous studies was acceptable to excellent with Cronbach's alphas between 0.68 and 0.97. (e.g., Koch et al., 2007, 2015), and factor analyses mostly yielding a general factor of “positivity vs. negativity.”

##### Body self efficacy

Body Self Efficacy (BSE) was assessed with the BSE-scale (Fuchs and Koch, 2014). The validated German version of this questionnaire contains a 10 item scale measuring the self-perception of bodily abilities (“I can's;” Husserl, 1952) with items such as “My body is flexible,” “My body feels like in pieces,” etc., on rating scales from “0” (“does not apply at all”) to “5” (“applies exactly”). In former studies, the internal consistency of the German version of the scale was Cronbach's alpha = 0.75 in a student sample, and Cronbach's alpha = 0.83 in a clinical sample (Kelbel, 2013; Fuchs and Koch, 2014).

##### The BSE-beauty subscale

The BSE-beauty subscale consisted of the two items “My movements are beautiful” and “I can move elegantly/with grace.” We looked at this subscale separately, because in working with PD patients, we became increasingly aware that art-based intervention have additional therapeutic factors other than functional and classical psychological ones.

##### Expectancies

Patients' expectancies of the therapy outcome were measured using the Credibility Expectancy Questionnaire (CEQ; Devilly and Borkovec, 2000) with four items on cognitive expectancy (credibility), e.g., “How logical does the therapy offered to you seem?” on a rating-scale from “1” “not at all” to “10” “very much,” and four items on affective expectancy, e.g., “How much do you really feel that the therapy will help you to reduce your symptoms?”). In previous studies (e.g., Devilly and Borkovec, 2000) high internal consistencies were found for both the cognitive (Cronbach's alpha = 0.86) as well as the affective expectancy factor (Cronbach's alpha = 0.90). The English version of the CEQ was translated into German for the purpose of this study by co-author Judith Raeke.

##### Therapeutic factors of arts therapies in PD related to the aesthetic experience

The scale on therapeutic factors of arts therapies in PD (Mergheim, 2015; see Appendix C in Supplementary Material) was composed on the basis of face validity of the symptoms and needs of the PD patients and the assumed aspects of the aesthetic experience such as beauty, flow, happiness, unity with self and unison with partner. It further contained items on pleasure/joy, expressiveness, and communication fluency.

*Feasibility* of appropriateness and acceptance of the workshop format was assessed by observations, conversations with participants, as well as *short interviews* with volunteering patients (collected by a research assistant; and for a radio report after the second workshop).

### Statistical analysis

Outcome measures of the exploratory study were analyzed with a *t*-test for paired samples for pre–post differences with time as the factor using SPSS (version 23.0). The alpha-level was set to 0.05. After Bonferroni correction the new Alpha-level for the primary outcome was 0.01, and for the secondary outcome 0.008.

## Results

### Feasibility and acceptance

Feasibility of the intervention was evidenced (a) by the fact that merely one participant had to sit out from the intervention for physical reasons, (b) by observations of an increase in patients' positive affect, (c) by participant utterances in brief interviews (see below), and (d) by the fact that we received continued requests for more workshop offers from participants.

Participants profited regardless of their aims. A recently diagnosed woman had the aim “I want to fight the stiffness of the limbs and the difficulties with balance with movements that keep me as mobile as possible; my kids are 13 and 17 years old and still need me; that requires a certain speed in everyday life; I hope to keep up with them and be able to share their tempo” (age 52, for 3 years diagnosed with PD); and an older lady: “I am shaky and slow, and lately it has been getting worse; here I am fighting to keep what I have” (age 79, for 27 years diagnosed with PD).

Despite the challenge of the workshop for many participants, evaluations of the *Tango for PD* intervention were positive “I finally can breath again,” “I feel happier, more free, and mobile” “When I arrived I was totally down…that has changed,” or “There were more changes happening than I expected. I feel good and feel ready to continue,” “The workshop has been fun and inspired me to continue, I want to do more,” “This wonderful workshop has caused great joy, I want to continue in any case,” or “Even though it was physically demanding, it worked for me.” No negative voices were recorded in the interviews, yet since only volunteers were interviewed, self-selection bias needs to be accounted for.

The final statement of the mother with the two children from above was “When one realizes that it becomes increasingly more difficult to move, there is a high probability that one withdraws. The prescribed exercise is often an unloved duty. But here in the tango workshop, everybody is in a similar situation. This takes away the achievement pressure. Music and dance were completely relaxing, and the movement became increasingly easy; for a moment, I had totally forgotten that I am actually sick.”

### Exploratory study

#### Primary outcomes

**Figure 3:**
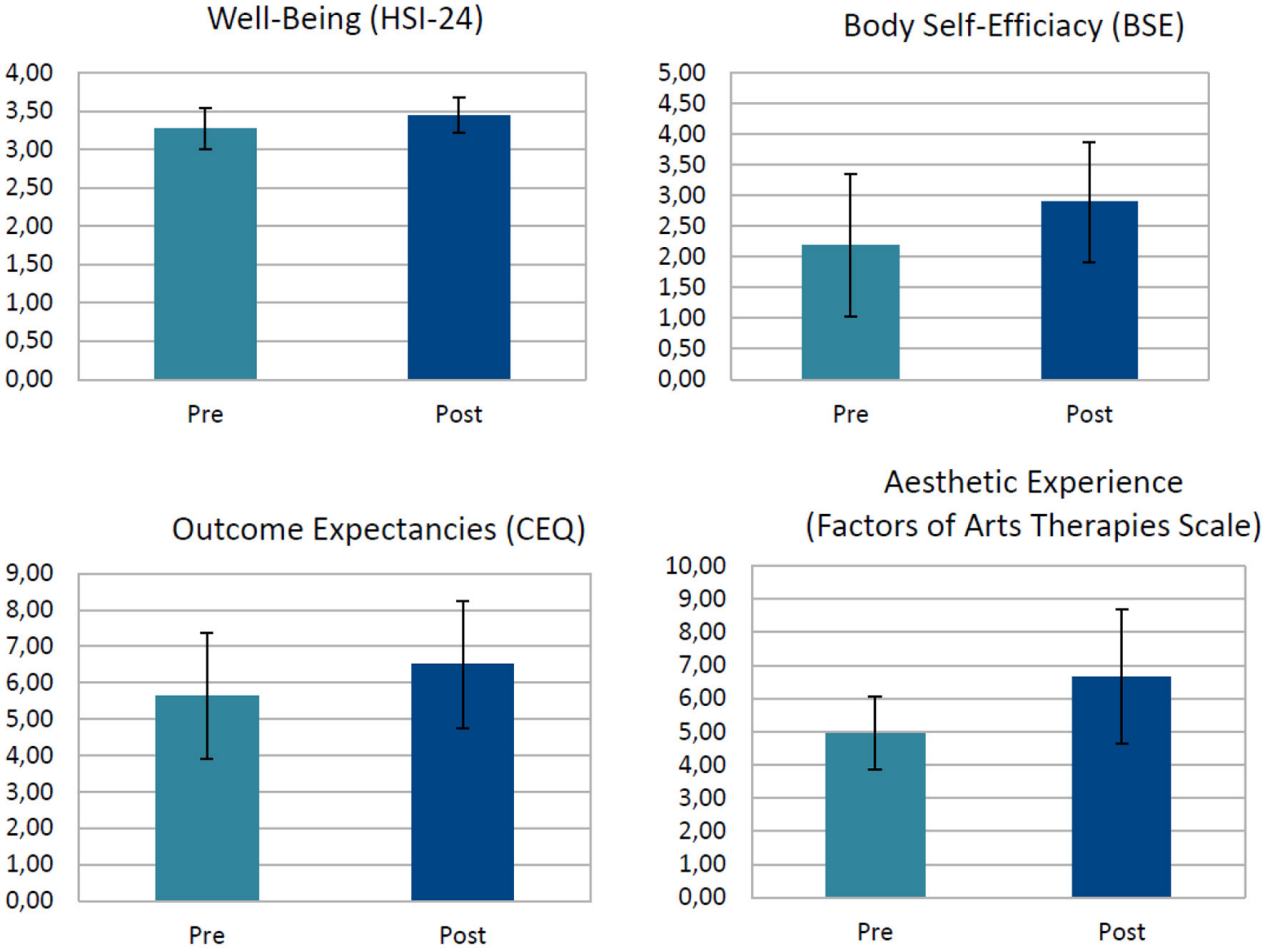
**Main effects of a single ***Tango for Parkinson*** intervention**. Well-being was measured on a 6-point scale from, CEQ on a 9-point scale, Body Self-Efficacy on a 6-point scale, and Therapeutic Factors of Arts Therapies on a 10-point scale; HSI-24; Heidelberg State Inventory, CEQ, Cognitive Expectancy Questionnaire.

In the post-test participants showed significantly improved scores on well-being, body self-efficacy and the cognitive aspect of outcome expectancy (see Table 2; Figure 3). Controlling for workshop group yielded no significant differences between groups regarding the outcome.

**Table 2:** **Primary outcomes: effects of Tango for PD on health-related psychological outcomes**.

		***N***	***M***	***SD***	***t***	***df***	***p***	***d***
Well-being (HSI-24)	Pre	34	3.27	0.27				
	Post	34	3.45	0.23	–3.73	33	0.001	0.69
Cognitive expectancies (CEQ)	Pre	34	5.96	1.61				
	Post	34	6.85	1.61	–4.01	33	0.000	0.55
Affective expectancies (CEQ)	Pre	34	5.35	1.85				
	Post	34	6.16	1.89	–3.31	33	0.002	0.44
Body self-efficacy (BSE)	Pre	34	2.19	1.17				
	Post	34	2.89	0.98	–3.59	33	0.001	0.65
BSE-beauty	Pre	29	2.00	1.31				
	Post	29	2.79	1.11	–2.81	28	0.009	0.66

*Descriptive results: Means (M), standard deviation (SD), and sample size (N); higher post-test scores represent improvement; Inferential statistics: t-value (t), degrees of freedom (df), probability-value (p), and effect size (Cohen's d); Design: pre–post within-group comparison; based on Bonferroni-correction values of p < 0.01 reflect significant differences*.

The changes of means from pre- to post-test scores increased significantly across the four measures. Well-being *t*_(33)_ = −3.73; *p* = 0.001, *d* = 0.69, cognitive outcome expectancy *t*_(33)_ = −4.02; *p* = 0.000, *d* = 0.55, affective outcome expectancy *t*_(33)_ = −3.31; *p* = 0.002, *d* = 0.44, body self-efficacy *t*_(33)_ = −3.59; *p* = 0.001, *d* = 0.65, and the beauty aspect of BSE *t*_(28)_ = −2.81; *p* = 0.009. *d* = 0.66, all improved beyond the critical alpha of 0.01 (effects sizes are Cohen's *d*s).

#### Secondary outcomes

Given that the aesthetic experience is assumed to be an important active factor (mediating mechanism; Koch, 2016) in the arts therapies and the related input of our continuous groups' members, and the fact that the BSE subscale of beauty (with two items) improved with *t*_(28)_ = −2.81; *p* = 0.009. *d* = 0.66, we constructed a measure to investigate the aesthetic experience in PD more closely. The therapeutic factors scale of eight items reflects central hypothesized active factors in arts therapies related to the aesthetic experience particularly geared toward PD patients, such as experienced beauty, pleasure/joy, happiness, expressiveness, fluency of movement and speech, body–mind unity, and unison with the partner. Participants reported an increase in the aesthetic experience after the tango workshop (see Table 3):

**Table 3:** **Secondary outcomes: therapeutic factors of arts therapies and effects of aesthetic experience in Tango for PD**.

		***N***	***M***	***SD***	***t***	***df***	***p***	***d***
Happiness	Pre	11	5.18	1.722				
	Post	11	7.09	2.119	–3.60	10	0.005	1.09
Beauty	Pre	11	5.09	1.578				
	Post	11	6.73	1.902	–2.84	10	0.018	0.99
Joy/Pleasure	Pre	11	5.27	1.679				
	Post	11	6.64	2.378	–2.19	10	0.053	0.94
Emotional expression	Pre	11	5.36	1.567				
	Post	11	6.82	2.316	–2.67	10	0.024	0.67
Flow of speech	Pre	11	5.27	1.902				
	Post	11	6.73	2.453	–2.14	10	0.058	0.75
Flow of movement	Pre	11	3.82	1.168				
	Post	11	6.36	1.912	–4.08	10	0.002	0.67
Body–mind unity	Pre	11	4.64	1.748				
	Post	11	6.55	1.864	–2.81	10	0.019	1.65
Unison with partner	Pre	11	5.00	2.569				
	Post	11	6.73	1.954	–2.73	10	0.021	1.06
**Total score**	**Pre**	**11**	**4.95**	**1.11**				
	**Post**	**11**	**6.66**	**2.02**	**–3.82**	**10**	**0.003**	**0.76**

*First results of the scale on Therapeutic Factors in Arts Therapies for PD, created to capture the aesthetic experience and other active factors (Appendix D in Supplementary Material); Descriptive Results: Means (M), standard deviation (SD), and sample size (N); higher post-test scores represent improvement; Inferential statistics: t-value (t), degrees of freedom (df), probability-value (p), and effect size (Cohen's d); based on Bonferroni-correction values <0.008 reflect significant differences; items 1–3 showed significant correlations among each other*.

Happiness increased after the tango intervention *t*_(10)_ = −3.60, *p* = 0.005, *d* = 0.99, and experienced beauty *t*_(10)_ = −2.84, *p* = 0.018, *d* = 0.94; so did other active factors of arts therapies related to aesthetic experience, such emotional expression *t*_(10)_ = −2.67, *p* = 0.024, *d* = 0.75, flow of movement *t*_(10)_ = −4.08, *p* = 0.002, *d* = 1.65, body–mind unity *t*_(10)_ = −2.81, *p* = 0.019, *d* = 1.06, and unison with partner *t*_(10)_ = −2.73, *p* = 0.021, *d* = 0.76. Joy/pleasure *t*_(10)_ = −2.19, *p* = 0.053, *d* = 0.67, and flow of speech *t*_(10)_ = −2.14, *p* = 0.058, *d* = 0.67 suggest a tendency in the expected direction; the effect sizes provide arguments for further testing. The total score of the *Therapeutic Factors of Arts Therapies for PD Scale* showed a significant increase with *t*_(10)_ = −3.82, *p* = 0.003, which was beyond the critical alpha of 0.008. The effect size of the entire scale was Cohen's *d* = 1.09.

Because the results of the *Therapeutic Factors of Arts Therapies for PD Scale* rest on a subsample of 11 patients only, they need to be interpreted with according caution, but can provide first ideas for follow-up studies.

## Discussion

We tested the feasibility of a single *Tango for Parkinson* intervention for measuring effects on health-related psychological outcomes. After the intervention, we observed increased well-being, body self-efficacy and outcome expectancies; our exploratory results are in line with prior and present studies suggesting positive effects of dance on psychological outcomes (Koch et al., 2014). Results suggest feasibility of the single tango intervention regarding the appropriateness and acceptance of the workshop format for PD patients and the sensitivity of the chosen measures in such a context. The intervention can now be included into a controlled design to compare, for example, Argentine Tango and other enjoyable non-dance movement interventions; a design that would allow to further identify/control for aesthetic experience.

The acceptance of patients may be particularly high, because Tango does not only address the physical and social aspects of the disease, but also the psychological co-morbidities of it and the beauty aspect that we find in cultural and arts-based interventions. It may thus be experienced as more holistic, affecting the entire person, as opposed to more functional techniques. One could rightfully ask, whether cultural techniques, in general, should thus be attributed a more central role in rehabilitation programs. The exploratory findings of the study were also encouraging, particularly considering the small—yet appropriately powered—sample size and the short duration of the intervention. However, careful interpretation of the results is warranted, because of the non-controlled character of the study. The study suggests that the tango intervention may positively influence well-being, patient's cognitive expectancies, and body self-efficacy. The suggested increase in *well-being* with a medium effect size is in line with prior findings of effects of dance movement therapy on other patient groups such as depressed patients (Koch et al., 2007), or subclinical samples. Movement in a protected setting seems to generally stimulate positive affect, vitality, and coping, and to decrease tension, depressed affect, and anxiety. In this study, particularly positive affect, vitality and coping increased. We would like to encourage other groups to make use of measures such as the HSI-24 that cover mental health variables, since depressed and anxious affect—often poorly recognized—are in many cases influential comorbidities of PD.

This is the one of the first attempts to address the importance of patients' *expectancies* in dance movement therapy. Cognitive and affective expectancies increased after the intervention, leading to the assumption that the interest and motivation to continue the activity was present in the participating patients (which was also perceptible from repeated requests for more workshops of patients, who lived too far away to come traveling to the regular groups). Interestingly, the data suggests that patients with initially higher expectations showed a higher increase in well-being and body self-efficacy, and less negative affect than participants with lower initial expectancies of therapy outcome. Although this was not the focus of the study, it does highlight the importance of the expectancy construct for other studies exploring group differences, especially where the initial expectancies could explain some differences of the later therapy outcome and attrition (Ametrano, 2011).

*Body self-efficacy* increased in the PD patients after the intervention. Even though or maybe because of the fact that patients verbally reported the intervention to have been a challenge for them, the feeling to have mastered the challenge could have been one contributing factor to this increase of the belief in one's own bodily skills. An enactive approach supports this argument, putting agency at the base of identity and health. The embodied-enactive approach is more suited than a classic cognitivist account for explaining effect of Tango on Parkinson's disease, in that it takes into account bodily self-regulation, and action-perception coupling; those processes are characterized by multiple feedback relations, and cannot be captured by linear input–output schemata.

The intervention can be as short as 1.5 h and already show an effect on psychological outcomes. This extends the assumption of Earhart (2009), Hackney et al. (2007), or Duncan and Earhart (2012) that short-term interventions start at 6 weeks, possibly owed to the fact that these researchers are physical therapists focusing on functional changes; whereas psychological changes, emotional, as well as motivational, can sometimes come about much faster. For measuring psychological change, our intervention of only 1.5 h was feasible, acceptable and appropriate for the patient group.

### Experiencing beauty

In arts therapies, we assume that the aesthetic experience is an important health predictor. In tango, the patients not only practice functional skills such as walking backward over an extended time, turning, initiating, and stopping, but also experience the pleasure of the music, of the company of their partner, and of the dance as a holistic experience *per se*. In a moment of aesthetic experience, I feel alive, I feel my body in unison with my mind (thoughts and feelings are in a heightened congruency), and I may experience my own body or movements as beautiful. These moments of aesthetic experience emerge in a complex pattern and are thus difficult to capture. Yet, they are crucial to understanding the workings of the arts therapies, such as dance movement therapy (see Box 1).

Box 1 **Arts and health: What is assumed as healing factors across the arts?**

**Clusters of therapeutic factors/active factors in arts therapies (Koch, 2016):**

**(a) Hedonism:** Art for pleasure and play → probing and enacting (future and past), “as if” space

**(b) Aesthetics:** Art for beauty and authentic expression → integration/body–mind unity

**(c) (Non-verbal) Meaning Making:** Art for symbolizing and communicating, art for being seen → imagery

**(d) Transitional Support:** Art for shelter, art for being seen (as beautiful), art for mastery, art for closure → rituals, mirroring

**(e) Productivity, Creation:** Art for resilience and self-efficacy (strength and control), art for leaving something behind → traces

Well-being and positive affect are important preconditions of a more *global cognitive processing* of stimuli (Gasper and Clore, 2002; Bless and Fiedler, 2006). They may challenge the patient away from more detailed processing and rumination to a more holistic processing style where unity of body and mind possibly can be experienced more easily. Positive outcome expectations and the increased experience of self-efficacy/being in control may additionally lead to a more global processing style (Gasper and Clore, 2002), this could in turn support a more holistic perception and an increased experience of the body–mind unity (as parts of the aesthetic experience; see results for the *body–mind-unity* item).

The experienced unity of body and mind may be “recalibrated” in movement therapy sessions (Edelhäuser et al., 2015; Heusser, 2015), and the unison with the partner, or the group in tango therapy may play an additional role in that readjustment. Tango therapy can therefore be seen as keeping the brain-body-environment system adaptive. Although PD patients are often slower and more rigid in their movements, attunement and kinesthetic empathy are still possible. It seems that the plasticity of the brain-body-environment system can be increased by challenging it via movement (see results for the *flow of movement* item). It is the task of the therapist to find the right music, to synchronize to movements and rhythms of the patient, and to provide the amount of scaffolding that matches the needs of the patients.

### Limitations

Limitations of the feasibility study are the small sample size. Yet, for the exploratory test, assuming an effect size of *d* = 0.44, a *p-*value of 0.05, and a power of 0.80, the required sample size equals exactly *N* = 34 patients (computed with G^*^Power, Faul et al., 2007). The biggest limitation of the exploratory test part of the study is its non-controlled character: all results reported here are merely suggestive and not conclusive. Moreover, the sample consisted of patients who were engaged in support groups and who were actively looking for alternative therapies, this could have contributed to higher levels of motivation and therapy expectancies compared to non-members of support groups. Moreover, we had no objective data other than self-report concerning patients' PD diagnoses, its severity and their medication. Further studies with a bigger sample should include those variables and ensure generalizability. During the tango intervention the participants were free to choose their dance partners (spouses or relatives, students, or other patients), it is unclear whether and how the partners or the number of partner changes influenced the results. Because we had no control group, other factors such as the structured group activity, the degree of physical activation, etc., may have influenced the results.

Another limitation concerns the slightly different situational factors at the different workshops, such as live music at the end of the first workshop and a slightly shortened session in the last workshop due to external conditions. It would be important for further studies to keep these conditions standardized. Moreover, because of certain motor difficulties such as a hand tremor some patients needed help from partners and relatives to fill out the questionnaires, this could have influenced the result with respect to social desirability. In general, there may have been social desirability and demand effects, which were probably one of the biggest problems of the studies. Since the study was uncontrolled, we cannot rule out that the increase in values after the intervention was merely due to the fact that we created a pleasant atmosphere, transmitted a caring attitude, or a firm belief in the effectiveness of the intervention, or alike. Transmitted beliefs additionally may have caused expectancy effects, for example trough the speech of the first author at the beginning of the workshops. However, the expectancy questionnaire did not show a decrease but an increase in expectancies after the intervention, providing evidence against the latter assumption. In sum, future research is called to replicate this study with one or more control groups using a randomized allocation of patients.

## Conclusions

This exploratory study led to some interesting starting points for future research. Feasibility of measuring health-related psychological variables from a single tango intervention was given. Yet, long-term interventions and randomized controlled trials (RCTs) using Tango Argentino should investigate both health-related psychological symptoms as well as motor symptoms and their interactions in order to improve therapies for persons with PD. In fact, PD-symptoms should be directly included on scales, which was not possible here, because of the limited time frame of our study. Research could then test, whether short-term interventions have stronger effects on psychological, and long-term interventions have stronger effects on physiological variables. Specificity of the Tango intervention needs to be further addressed: can other interventions do just the same, and if so, which ones? Equally important is the separation of contributing factors, such as the role of music, rhythm, psychological factors, preferences, etc. For example, Nombela et al. (2013) suggest that music facilitates activation of motor networks that bypass the disease-affected networks via cerebellum–thalamic–cortical circuitry. Therefore, it is important to objectify the musical, personal, and contextual variables that influence motor behavior in PD and other neurological diseases.

Future studies can investigate patients' expectancies as possible mediators to explain group differences, and can help to derive indications and contraindications for dance therapy with this specific population. Future studies with arts-based interventions should look at the additional outcomes of *depression, anxiety*, and *body image* changes, as well as the mediating factors of *rhythmic activity* (Sandel et al., 1993; Hackney et al., 2015, this volume), and *resonance* with self and other (Koch, 2011; Fuchs and Koch, 2014), in terms of *body feedback* (Koch, 2014), and *embodied intersubjectivity* (Fuchs and Koch, 2014).

To summarize, the study supports that a single dance movement therapy *Tango for PD* intervention is feasible for measuring changes on health-related psychological outcomes. It finds a positive relation between the tango intervention and the health-and adherence-related psychological outcomes of well-being, body self-efficacy, and outcome expectancies in PD patients, and identifies the potentially influential mediator of aesthetic experience. Due to the lack of a control group, results of this study are only suggestive, not conclusive. The usefulness of embodiment approaches and the role of the aesthetic experience, as a therapeutic factor of the arts therapies, is an aspect of *Tango for PD* that calls for further attention and investigation.

## Author contributions

All authors contributed substantially to this work. SK analyzed the data, is responsible for the model, and wrote the first article draft, SK, KM, and JR collected and analyzed the data of the first workshop, KM, NH, and JR collected the data of the second workshop, and KM, ER, and SK collected the data of the third workshop and wrote up the corresponding parts. KM connected the Tango for PD workshops with the model of embodied aesthetics in her Master's Thesis, CM and ER conducted the intervention, consulted on the manuscript from the dance therapy side, and wrote the intervention description, GD consulted and co-authored from the physiotherapy side, DM, JN, and TH consulted and co-authored from the music therapy side. DM and TH directed the institution under whose supervision the study was conducted. Authors discussed the results and commented on the manuscript at all stages.

### Conflict of interest statement

The authors declare that the research was conducted in the absence of any commercial or financial relationships that could be construed as a potential conflict of interest.

## Footnotes

^1^Embodied simulation theory has been put forward to explain bodily resonance in light of the discovery of a mirror mechanisms in the brain (Rizzolatti et al., 1996; Freedberg and Gallese, 2007).

## References

[B1] Abbruzzese G. Marchese R. Avanzino L. Pelosin E. (2016). Rehabilitation for Parkinson's disease: current outlook and future challenges. Parkinsonism Relat. Disord. 22(Suppl. 1), 60–64. 10.1016/j.parkreldis.2015.09.005 26360239

[B2] Allesch C. (2006). Einführung in die Psychologische Ästhetik [Introduction to Psychological Aesthetics]. Wien: Facultas.

[B3] Ametrano R. M. (2011). Patient Outcome Expectations and credibility Beliefs as Predictors of the Alliance and Treatment Outcome. Unpublished Thesis, University of Massachusetts.

[B4] Ashburn A. Roberts L. Pickering R. Roberts H. C. Wiles R. Kunkel D.. (2014). A design to investigate the feasibility and effects of partnered ballroom dancing on people with Parkinson disease: randomized controlled trial protocol. JMIR Res. Protoc. 3, e34. 10.2196/resprot.3184 25051989 PMC4137144

[B5] Baumgarten A. (1750/2007). Ästhetik. Hamburg: Meiner (Originally published in 1750).

[B6] Blandy L. M. Beevers W. A. Fitzmaurice K. Morris M. E. (2015). Therapeutic Argentine tango dancing for people with mild Parkinson's disease: a feasibility study. Front. Neurol. 6:122. 10.3389/fneur.2015.00122 26074873 PMC4445309

[B7] Bless H. Fiedler K. (2006). Mood and the regulation of information processing and behavior, in Hearts and Minds: Affective Influences on Social Cognition and Behavior, eds Forgas J. P. Williams K. D. van Hippel W. (New York, NY: Psychology Press), 65–84.

[B8] Chagas M. Linares I. Garcia G. Hallak J. Tumas V. Crippa J. (2013). Neuroimaging of depression in Parkinson's disease: a review. Int. Psychogeriatr. 25, 1953–1961. 10.1017/S1041610213001427 23992107

[B9] Chatterjee A. Vartanian O. (2014). Neuroaesthetics. Trends Cogn. Sci. 18, 370–375. 10.1016/j.tics.2014.03.003 24768244

[B10] DeDreu M. J. Van Der Wilk A. S. D. Poppe E. Kwakkel G. Van Wegen E. E. H. (2012). Rehabilitation, exercise therapy and music in patients with Parkinson's disease: a meta-analysis of the effects of music-based movement therapy on walking ability, balance and quality of life. Parkinson. Relat. Disord. 18, 114–119. 10.1016/S1353-8020(11)70036-0 22166406

[B11] DeJaegher H. DiPaolo E. (2007). Participatory sense-making: an enactive approach to social cognition. Phenomenol. Cogn. Sci. 6, 475–507.

[B12] Devilly G. J. Borkovec T. D. (2000). Psychometric properties of the credibility/expectancy questionnaire. J. Behav. Ther. Exp. Psychiatry 31, 73–86. 10.1016/S0005-7916(00)00012-4 11132119

[B13] Dibble L. E. Addison O. Papa E. (2009). The effects of exercise on balance in persons with Parkinson's disease: a systematic review across the disability spectrum. J. Neurol. Physiol. Ther. 33, 14–26. 10.1097/NPT.0b013e3181990fcc 19265767

[B14] Duncan R. P. Earhart G. M. (2012). Randomized controlled trial of community-based dancing to modify disease progression in Parkinson disease. Neurorehabil. Neural Repair 26, 132–143. 10.1177/1545968311421614 21959675

[B15] Duncan R. P. Earhart G. M. (2014). Are the effects of community-based dance on parkinson disease severity, balance, and functional mobility reduced with time? A 2-year prospective pilot study. J. Alternat. Complement. Med. 20, 757–763. 10.1089/acm.2012.0774 25192393

[B16] Earhart G. M. (2009). Dance as therapy for individuals with parkinson disease. Eur. J. Phys. Rehabil. Med. 45, 231–238. 19532110 PMC2780534

[B17] Edelhäuser F. Minnerop A. Trapp B. Büssing A. Cysarz D. (2015). Eurythmy therapy increases specific oscillations of heart rate variability. BMC Complement. Altern. Med. 15:167. 10.1186/s12906-015-0684-6 26047615 PMC4457978

[B18] Faul F. Erdfelder E. Lang A.-G. Buchner A. (2007). G*Power 3: A flexible statistical power analysis program for the social, behavioral, and biomedical sciences. Behav. Res. Methods 39, 175–191. 17695343 10.3758/bf03193146

[B19] Fisher B. E. Wu A. D. Salem G. J. Song J. Lin C.-H. Yip J.. (2008). The effect of exercise training in improving motor performance and corticomotor excitability in people with early Parkinson's disease. Arch. Physiother. Med. Rehabil. 89, 1221–1229. 10.1016/j.apmr.2008.01.013 18534554 PMC2989816

[B20] Foster E. R. Golden L. Duncan R. P. Earhart G. M. (2013). Community-based Argentine tango dance program is associated with increased activity participation among individuals with Parkinson's disease. Arch. Phys. Med. Rehabil. 94, 240–249. 10.1016/j.apmr.2012.07.028 22902795 PMC3557593

[B21] Freedberg D. Gallese V. (2007). Motion, emotion and empathy in esthetic experience. Trends Cogn. Sci. 11, 197–203. 10.1016/j.tics.2007.02.003 17347026

[B22] Fuchs T. Koch S. C. (2014). Embodied affectivity: on moving and being moved. Front. Psychol. 5:508. 10.3389/fpsyg.2014.00508 24936191 PMC4047516

[B23] Fuchs T. Schlimme J. (2009). Embodiment and psychopathology: a phenomenological perspective. Curr. Opin. Psychiatry 22, 570–575. 10.1097/YCO.0b013e3283318e5c 19730373

[B24] Gallese V. (2001). The ‘shared manifold’ hypothesis: from mirror neurons to empathy. J. Conscious. Stud. 8, 33–50.

[B25] Gasper K. Clore G. (2002). Attending to the big picture: mood and global versus local processing of visual information. Psychol. Sci. 13, 34–40. 10.1111/1467-9280.00406 11892776

[B26] Gibson J. J. (1966). The Senses Considered as Perceptual Systems. Boston, MA: Houghton Mifflin.

[B27] Gifford R. (1997). Environmental Psychology: Principles and Practices. Needham Heights, MT: Allyn and Bacon.

[B28] Goodill S. W. (2006). Dance/movement therapy for populations living with medical illness, in Advances in Dance/Movement Therapy. Theoretical Perspectives and Empirical Findings, eds Koch S. C. Brauninger I. (Berlin: Logos), 52–60.

[B29] Goodwin V. A. Richards S. H. Henley W. Ewings P. Taylor A. H. Campbell J. L. (2011). An exercise intervention to prevent falls in people with Parkinson's disease: a pragmatic randomised controlled trial. J. Neurol. Neurosurg. Psychiatry 82, 1232–1238. 10.1136/jnnp-2011-300919 21856692

[B30] Goodwin V. A. Richards S. H. Taylor R. S. Taylor A. H. Campbell J. L. (2008). The effectiveness of exercise interventions for people with Parkinson's disease: a systematic review and meta-analysis. Mov. Disord. 23, 631–640. 10.1002/mds.21922 18181210

[B31] Hackney M. E. Bennett C. G. (2014). Dance therapy for individuals with Parkinson's disease: improving quality of life. J. Parkinson. Restless Legs Syndr. 14, 17–25. 10.2147/JPRLS.S40042

[B32] Hackney M. E. Earhart G. M. (2009). Effects of dance on movement control in Parkinson's disease: a comparison of Argentine tango and American ballroom. J. Rehabil. Med. 41, 475–481. 10.2340/16501977-0362 19479161 PMC2688709

[B33] Hackney M. E. Earhart G. M. (2010). Effects of dance on gait and balance in Parkinson's disease: a comparison of partnered and non-partnered dance movement. Neurorehabil. Neural. Rep. 24, 384–392. 10.1177/1545968309353329 PMC2900796 20008820

[B34] Hackney M. E. Kantorovich S. Levin R. Earhart G. M. (2007). Effects of tango on functional mobility in Parkinson's disaese: a preliminary study. J. Neurol. Phys. Ther. 31, 173–179. 10.1097/NPT.0b013e31815ce78b 18172414

[B35] Hackney M. E. Lee H. L. Battisto J. Crosson B. McGregor K. M. (2015). Context-dependent neural activation: internally and externally guided rhythmic lower limb movement in individuals with and without neurodegenerative disease. Front. Neurol. 6:251. 10.3389/fneur.2015.00251 26696952 PMC4667008

[B36] Haken H. (2010). Information and Self-organization: A Macroscopic Approach to Complex Systems, 3rd Edn. Berlin: Springer.

[B37] Hashimoto H. Takabatake S. Miyaguchi H. Nakanishi H. Naitou Y. (2015). Effects of dance on motor functions, cognitive functions, and mental symptoms of Parkinson's disease: a quasi-randomized pilot trial. Complement. Ther. Med. 23, 210–219. 10.1016/j.ctim.2015.01.010 25847558

[B38] Heusser P. (2015). Vortrag Zur Eröffnung Des Forschungsinstituts Für Künstlerische Therapien [Presentation at the Opening of the Research Institute for Creative Arts Therapies] (RIArT). Alfter: Alanus University.

[B39] Husserl E. (1952). Ideen zu einer reinen Phänomenologie und phänomenologischen Philosophie II. Husserliana, Bd. 4. Den Haag: Nijhoff.

[B40] Hwang P. W. Braun K. L. (2015). The effectiveness of dance interventions to improve older adults' health: a systematic review. Altern. Ther. Health Med. 21, 64–70. 26393993 PMC5491389

[B41] Johansson P. Høglend P. Hersoug A. (2011). Therapeutic alliance mediates the effect of patient expectancy in dynamic psychotherapy. Br. J. Clin. Psychol. 50, 283–297. 10.1348/014466510X517406 21810107

[B42] Kattenstroth J. C. Kalisch T. Holt S. Tegenthoff M. Dinse H. R. (2013). Six months of dance intervention enhances postural, sensorimotor, and cognitive performance in elderly without affecting cardio-respiratory functions. Front. Aging Neurosci. 5:5. 10.3389/fnagi.2013.00005 23447455 PMC3581819

[B43] Kelbel J. (2013). Embodiment – Validierung der Fragebögen zur “Body-Self Efficacy und Embodied Intersubjectivity.” Unpublished Thesis, Heidelberg, University of Heidelberg.

[B44] Kelso J. A. S. (1995). Dynamic Patterns: The Self-Organization of Brain and Behavior. Cambridge: MIT Press.

[B45] Koch S. C. (2011). Basic body rhythms and embodied intercorporality: from individual to interpersonal movement feedback, in The Implications of Embodiment: Cognition and Communication, eds Tschacher W. Bergomi C. (Exeter: Imprint Academic) 151–171.

[B46] Koch S. C. (2014). Rhythm is it: effects of dynamic body-feedback on affect attitudes and cognition. Front. Psychol. 5:537 10.3389/fpsyg.2014.00537 PMC4051267 24959153

[B47] Koch S. C. (2016). Arts and Health: Active Factors of Arts Therapies and a Theory Framework of Embodied Aesthetics. Under Review at The Arts in Psychotherapy.

[B48] Koch S. C. Fischman D. (2011). Embodied enactive dance therapy. Am. J. Dance Ther. 33, 57–72. 10.1007/s10465-011-9108-4

[B49] Koch S. C. Fuchs T. (2011). Embodied arts therapies. Arts Psychother. 38, 276–280. 10.1016/j.aip.2011.08.007

[B50] Koch S. C. Kunz T. Lykou S. Cruz R. (2014). Effects of dance and dance movement therapy on health-related psychological outcomes: A meta-analysis. Arts Psychother. 41, 46–64. 10.1016/j.aip.2013.10.004

[B51] Koch S. C. Morlinghaus K. Fuchs T. (2007). The joy dance: specific effects of a single dance intervention on psychiatric patients with depression. Arts Psychother. 34, 340–349. 10.1016/j.aip.2007.07.001

[B52] Koch S. C. Steinhage A. Haller K. Kende P. Ostermann T. Chyle F. (2015). Breaking barriers: evaluating and arts-based emotion regulation program in prison. Arts Psychother. 42, 41–49. 10.1016/j.aip.2014.10.008

[B53] Kreutz G. Quiroga Murcia C. (2015). Gesundheitliche aspekte des tanzens [Health aspects of dancing], in Musik und Medizin. Chancen für Therapie, Prävention und Bildung [Music and Medicine. Chances for Therapy, Prevention and Ducation], eds Bernatzky G. Kreutz G. (Wien: Springer), 285–302.

[B54] Lakoff G. Johnson M. (1999). Philosophy in the Flesh: The Embodied Mind and its Challenge to Western Thought. New York, NY: Basic Books.

[B55] Leder H. Belke B. Oeberst A. Augustin D. (2004). A model of aesthetic appreciation and aesthetic judgements. British J. Psychol. 95, 489–508. 10.1348/0007126042369811 15527534

[B56] Leder H. Nadal M. (2014). Ten years of a model of aesthetic appreciation and aesthetic judgments: the aesthetic episode—developments and challenges in empirical aesthetics. British J. Psychol. 105, 443–464. 10.1111/bjop.12084 25280118

[B57] Lewis C. Annett L. E. Davenport S. Hall A. A. Lovatt P. (2016). Mood changes following social dance sessions in people with Parkinson's disease. J. Health Psychol. 21, 483–492. 10.1177/1359105314529681 24752558

[B58] Lindenbach D. Bishop C. (2013). Critical involvement of the motor cortex in the pathophysiology and treatment of Parkinson's disease. Neurosci. Biobehav. Rev. 37(10 Pt 2), 2737–2750. 10.1016/j.neubiorev.2013.09.008 24113323 PMC3859864

[B59] Lötzke D. Ostermann T. Büssing A. (2015). Argentine tango in Parkinson disease – a systematic review and meta-analysis. BMC Neurol. 15:226. 10.1186/s12883-015-0484-0 26542475 PMC4636067

[B60] Mainka S. (2015). Music stimulates muscles, mind, and feelings in one go. Front. Psychol. 6:1547. 10.3389/fpsyg.2015.01547 26500596 PMC4597192

[B61] Mandelbaum R. Lo A. C. (2014). Examining dance as an intervention in Parkinson's disease: a systematic review. Am. J. Dance Ther. 36, 160–175. 10.1007/s10465-014-9181-6

[B62] Mannheim E. Weis J. (2006). Dance/Movement therapy with cancer patients. Evaluation of process and outcome parameters, in Advances in Dance/Movement Therapy. Theoretical Perspectives and Empirical Findings, eds Koch S. C. Bräuninger I. (Berlin: Logos), 61–72.

[B63] Martindale C. (1984). The pleasures of thought: a theory of cognitive hedonics. J. Mind Behav. 5, 49–80.

[B64] McKee K. E. Hackney M. E. (2013). The effects of adapted tango on spatial cognition and disease severity in Parkinson's disease. J. Mot. Behav. 45, 519–529. 10.1080/00222895.2013.834288 24116748 PMC3864026

[B65] McNeely M. E. Duncan R. P. Earhart G. M. (2015). A comparison of dance interventions in people with Parkinson disease and older adults. Maturitas 81, 10–16. 10.1016/j.maturitas.2015.02.007 25771040 PMC4497370

[B66] Mergheim K. (2015). Experiencing Beauty. Der heilende Faktor ästhetischer Erfahrung in den künstlerischen Therapien und seine Relevanz bei Parkinson. Unpublished Master's Thesis, Heidelberg, SRH University Heidelberg.

[B67] Merleau-Ponty M. (1962). Phenomenology of Perception. Transl. by Colin Smith. London: Routledge.

[B68] Merleau-Ponty M. (1964). Eye and Mind, in The Primacy of Perception. Transl by Dallery C. ed Edie J. (Evanston: Northwestern University Press), 159–190.

[B69] Michalak J. Rohde K. Troje N. F. (2015). How we walk affects what we remember: gait modifications through biofeedback change negative affective memory bias. J. Behav. Ther. Exp. Psychiatry 46, 121–125. 10.1016/j.jbtep.2014.09.004 25310681

[B70] Morris M. E. Martin C. L. Schenkman M. L. (2010). Striding out with Parkinson disease: evidence-based physical therapy for gait disorders. Phys. Ther. 90, 280–288. 10.2522/ptj.20090091 20022998 PMC2816030

[B71] Niedenthal P. M. Barsalou L. W. Winkielman P. Krauth-Gruber S. Ric F. (2005). Embodiment in attitudes, social perception, and emotion. Pers. Soc. Psychol. Rev. 9, 184–211. 10.1207/s15327957pspr0903_1 16083360

[B72] Nombela C. Hughes L. E. Owen A. M. Grahn J. A. (2013). Into the groove: can rhythm influence Parkinson's disease? Neurosci. Biobehav. Rev. 37(10 Pt 2), 2564–2570. 10.1016/j.neubiorev.2013.08.003 24012774

[B73] Quiroga Murcia C. Bongard S. Kreutz G. (2009). Emotional and neurohumoral responses to dancing tango argentino: the effects of music and partner. Music Med. 1, 14–21. 10.1177/1943862109335064

[B74] Quiroga Murcia C. Kreutz G. Clift S. Bongard S. (2010). Shall we dance? An exploration of the perceived benefits of dancing on well-being. Arts Health Int. J. Res. Policy Pract. 2, 149–163. 10.1080/17533010903488582

[B75] Ramachandran V. S. Hirstein W. (1999). The science of art. J. Conscious. Stud. 6(6 Pt 7), 15–51. 26649249

[B76] Ramsayer F. Tschacher W. (2011). Nonverbal synchrony in psychotherapy: coordinated body movement reflects relationship quality and outcome. J. Consult. Clin. Psychol. 79, 284–295. 10.1037/a0023419 21639608

[B77] Reber R. Schwarz N. Winkielman P. (2004). Processing fluency and aesthetic pleasure: Is beauty in the perceiver's processing experience? J. Mark. Res. 41, 151–165. 10.1207/s15327957pspr0804_3 15582859

[B78] Rizzolatti G. Fadiga L. Gallese V. Fogassi L. (1996). Premotor cortex and the recognition of motor actions. Cogn. Brain Res. 3, 131–141. 8713554 10.1016/0926-6410(95)00038-0

[B79] Salvatore S. Tschacher W. Gelo O. C. G. Koch S. C. (2015). Editorial: dynamic systems theory and embodiment in psychotherapy research. A new look at process and outcome. Front. Psychol. 6:914. 10.1016/j.jpolmod.2015.03.002 26191023 PMC4486829

[B80] Sandel S. Chaiklin S. Lohn A. (1993). Foundations of Dance/Movement Therapy: The Life and Work of Marian Chace. Columbia, MD: American Dance Therapy Association.

[B81] Scherer K. R. Wallbott H. G. (1985). Hand movement quality: a neglected aspect of nonverbal behavior in clinical judgment and person perception. J. Clin. Psychol. 41, 345–359. 3998157 10.1002/1097-4679(198505)41:3<345::aid-jclp2270410307>3.0.co;2-9

[B82] Schiavio A. Altenmüller E. (2015). Exploring music-based rehabilitation for Parkinsonism through embodied cognitive science. Front. Neurol. 6:217. 10.3389/fneur.2015.00217 26539155 PMC4609849

[B83] Schwarzer R. Warner L. M. (2013). Perceived self-efficacy and its relationship to resilience, in Resilience in Children, Adolescents, and Adults: Translating Research Into Practice, eds Prince-Embury S. Saklofske D. H. (New York, NY: Springer) 139–150.

[B84] Shanahan J. Morris M. E. Bhriain O. N. Saunders J. Clifford A. M. (2014). Dance for people with Parkinson's disease: what is the evidence telling us? Arch. Phys. Med. Rehabil. 96, 141–153. 10.1016/j.apmr.2014.08.017 25223491

[B85] Sharp K. Hewitt J. (2014). Dance as an intervention for people with Parkinson's Disease: a systematic review and meta-analysis. Neurosci. Biobehav. Rev. 47, 445–456. 10.1016/j.neubiorev.2014.09.009 25268548

[B86] Sheets-Johnstone M. (1999). The Primacy of Movement. Philadelphia, PA: John Benjamins.

[B87] Skorvanek M. Rosenberger J. Gdovinova Z. Nagyova I. Saeedian R. Groothoff J. W.. (2013). Apathy in elderly non-demented patients with Parkinson's disease: clinical determinants and relationship to quality of life. J. Geriatr. Psychiatry Neurol. 26, 237–243. 10.1177/0891988713500587 23970460

[B88] Sumec R. Filip P. Sheardová K. Bares M. (2015). Psychological benefits of nonpharmacological methods aimed for improving balance in Parkinson's disease: a systematic review. Behav. Neurol. 2015:620674. 10.1155/2015/620674 26236107 PMC4508472

[B89] Thelen E. Smith L. (1994). A Dynamic Systems Approach to the Development of Cognition and Action. Cambridge, MA: MIT Press.

[B90] Varela F. J. Thompson E. Rosch E. (1991). The Embodied Mind: Cognitive Science and Human Experience. Cambridge, MA: MIT Press.

[B91] von Weizsäcker V. (1940). Der Gestaltkreis. Theorie der Einheit von Wahrnehmen und Bewegen [The Gestalt-Circle. Theory of the Unity of Perception and Movement]. Leipzig: Thieme.

[B92] Wallbott H. G. (1982). Bewegungsstil und Bewegungsqualität [Movement Style and Movement Quality]. Weinheim: Beltz.

[B93] Wallbott H. G. (1990). Mimik im Kontext: Die Bedeutung verschiedener Informationskomponenten für das Erkennen von Emotionen. [Facial Expression in Context: The Meaning of Different Information Components for Emotion Recognition]. Goettingen: Hogrefe.

[B94] Wiedenhofer S. Hofinger S. Wagner K. Koch S. C. (2016). Active factors in dance/movement therapy I: effects of non-goal-orientation in movement on perceived stress, (body) self-efficacy, and well-being. Am. J. Dance Ther. [Epub ahead of print].

