# Supplementary material for: The Embodied Self in Parkinson's Disease: Feasibility of a Single Tango Intervention for Assessing Changes in Psychological Health Outcomes and Aesthetic Experience
Source: Front Neurosci. 2016 Jul 7;10:287. doi: 10.3389/fnins.2016.00287 (PMC4935674; doi:10.3389/fnins.2016.00287)
Supplement: Supplementary file 1 [file Presentation1.pdf]

## Appendix A: TANGO THERAPY for PD Patients in three workshops (2014 - 2015)

| | |
|---|---|
| 3 min. | **I - PRESENTATION** – names.<br>**Sitting position in circle** |
| 7 min. | **II – WARM UP**<br>**Sitting position, activation**<br><br>**BODY MOBILITY**<br><br>1 - Move the body's extremities: move one foot, two feet ... one hand. two hands ... head ... hand and foot ... hands and head ... feet. hands and head together.<br><br>2 - Work with support foot:<br><br>Raise heels supporting the weight on the toes, pushing the floor with the entire sole of the foot, raise the fingers supporting the weight on your heels.<br><br>Hanging weight from side to side. Hanging weight from side to side scrolling on the chair back and forth.<br><br>*** image used to oxygenate joints**<br><br>3- Move your shoulders up and down, in a circular way, together and apart.<br><br>4- Move the central axis (spine) dissociating the torso to the right and the left.<br><br>5- Patt your body from (arms, legs, neck, head), clap with your partners (interaction)<br><br>6- Move the torso: opening and closing arms hugging oneself.<br><br>*** use the image to release the light of a lantern in the center of the chest**<br><br>**III – THE DEVELOPMENT**<br><br>**BODY POSTURE**<br>**Standing position and round** |
| 2 min. | **1-** The individual positioning of tango: feet together, knees contact, closed legs, knees relaxed, tilted hip, torso upright, head up.<br><br>***Put a paper collar on the chest to symbolize the light of the lantern going forward** |
| 5 min. | 2- Move around the space maintaining posture. Give the lantern light for others.<br><br>Stop moving when the music silent and sort the individual positioning opening his arms and turning back drawing a big circle in the air. Move |

| | |
|---|---|
| | again. |
| | **<u>SHIFT WEIGHT AND RYTHM</u>** **<u>Standing position, holding hands, and round</u>** |
| 2 min. | 3- Mark the rhythm (4 times) with palms shifting weight from one leg to the other. |
| 3 min. | 4- Move laterally: a step towards the right (1 time) + shift weight (3 times). Change direction: a step to the left (1t) + shift weight (3t) |
| 2 min. | 5- Move forward: a step forward (1t) + shift weight (3t) + a step back (1t) + shift weight (3 t). |
| | **6-** All together: a step to the right (1t) + shift weight (3t) + a step forward (1t) + shift weight (3t) + a step back (1t) + shift weight (3t). Changing direction: start to the left side. |
| | **<u>WALK AND RHYTHM</u>** |
| 5 min. | 7- Walk forward treading on 4 times. Stop and shift weight (4t). Go forward (1t) + shift weight (3t). Go forward in time 1 and 3. Shift weight in time 2 and 4. |
| 3 min. | **8-** Make the same in couple with side hug. Explain axis prolongation. |
| | **<u>PAUSE</u>** |
| | **<u>TANGO: BASIC STEP 8 movements</u>** |
| 15 min. | 9- Split group into leaders and followers (male and female roles)<br>Teach the basic step till movement 5. Teach movements 6, 7, 8. |
| 15 min. | 10- Make the basic step with a partner. Change partners. |
| 15 min. | 11- Dance with live music. |
| | **IV – CLOSURE** |
| 8 min. | 12- Comments<br>**<u>Sitting position and round</u>** |
| 2 min. | **13-** Ritual: pat yourself on shoulder “well done!”, passing the “pulse energy” around the circle.<br>**<u>Standing position and round</u>** |
| | **Concept: Clarissa Machado / Eliane Riegner** |
