# Supplementary material for: The Embodied Self in Parkinson's Disease: Feasibility of a Single Tango Intervention for Assessing Changes in Psychological Health Outcomes and Aesthetic Experience
Source: Front Neurosci. 2016 Jul 7;10:287. doi: 10.3389/fnins.2016.00287 (PMC4935674; doi:10.3389/fnins.2016.00287)
Supplement: Supplementary file 2 [file Presentation2.pdf]

Appendix B: The Questionnaire (Primary Outcome Measures)

1. Credibility Expectancy Questionnaire (German version: Raeke, 2013; English version: Ametrano, 2011)

Wir bitten Sie im Folgenden anzukreuzen, wie sehr Sie jetzt gerade im Moment glauben, dass die Behandlung/Therapie Ihre Symptome reduzieren wird. Meistens gibt es zwei Aspekte: (1) Was man *glaubt*, was passieren wird und (2) was man *fühlt*, was passieren wird. Manchmal sind diese Aspekte sehr ähnlich, manchmal auch verschieden. Bitte beantworten Sie nun die folgenden Fragen. In Teil 1 beantworten Sie bitte, was Sie denken und in Teil 2, wie Sie fühlen.

Teil 1

1. Wie logisch finden Sie die Therapie zum jetzigen Zeitpunkt?

1 2 3 4 5 6 7 8 9

gar nicht logisch etwas logisch sehr logisch

2. Wie sehr denken Sie, dass die Therapie hilfreich ist, um Ihre Symptome zu reduzieren?

1 2 3 4 5 6 7 8 9

gar nicht hilfreich etwas hilfreich sehr hilfreich

3. Wie zuversichtlich wären Sie, diese Therapie einem Freund zu empfehlen, der ähnliche Probleme hat?

1 2 3 4 5 6 7 8 9

gar nicht zuversichtlich etwas zuversichtlich sehr zuversichtlich

4. Was denken Sie, um wieviel sich Ihre Symptome am Ende der Therapieeinheit verbessert haben werden?

0% 10% 20% 30% 40% 50% 60% 70% 80% 90% 100%

Teil 2

Für diesen zweiten Teil schließen Sie bitte kurz die Augen und spüren Sie in sich hinein, was Sie über die Therapie und deren möglichen Erfolg *fühlen*.

1. Wie sehr fühlen Sie, dass die Therapie hilfreich ist, um Ihre Symptome zu reduzieren?

1 2 3 4 5 6 7 8 9

gar nicht hilfreich etwas hilfreich sehr hilfreich

2. Was fühlen Sie, um wieviel sich Ihre Symptome am Ende der Therapieeinheit verbessert haben werden?

0% 10% 20% 30% 40% 50% 60% 70% 80% 90% 100%

2. Body Self-Efficacy-Scale (BSE; Koch, 2012; English version Fuchs & Koch, 2014)

Bitte geben Sie spontan die für Sie zutreffende Antwort an.

Hierbei bedeutet: 0 Trifft nicht zu — 5 Trifft genau zu

1. Ich kann mich gut bewegen.	0	1	2	3	4	5
2. Meine Bewegungen sind schön.	0	1	2	3	4	5
3. Mein Körper ist flexibel (beweglich).	0	1	2	3	4	5
4. Ich habe viele körperliche Mängel.	0	1	2	3	4	5
5. Mein Körper ist leblos und dumpf.	0	1	2	3	4	5
6. Ich kann leicht über ein Hindernis mittlerer Höhe springen.	0	1	2	3	4	5
7. Mein Körper fühlt sich an wie „in Stücken“.	0	1	2	3	4	5
8. Mein Körper fühlt sich oft an, als gehöre er nicht zu mir.	0	1	2	3	4	5
9. Ich kann mich elegant bewegen.	0	1	2	3	4	5
10. Ich kann mich in Bewegung ausdrücken.	0	1	2	3	4	5

3. **Heidelberg State Inventory** (HSI-24 unipolar version; former bipolar version by Koch, Morlinghaus & Fuchs, 2007)

	I momentarily feel…	Does not apply at all	Does not apply	Does rather not apply	Does rather apply	Does apply
TE1	Relaxed	☐	☐	☐	☐	☐
PA2	In a good mood	☐	☐	☐	☐	☐
AN3	Without worries	☐	☐	☐	☐	☐
TE4	Indulgent	☐	☐	☐	☐	☐
TE5	Tense	☐	☐	☐	☐	☐
PA6	In a bad mood	☐	☐	☐	☐	☐
DA7	Depressed	☐	☐	☐	☐	☐
AN8	Anxious	☐	☐	☐	☐	☐
DA9	Empty, without life	☐	☐	☐	☐	☐

CO10	Ready to deal with the world	☐	☐	☐	☐	☐
VI11	Full of energy	☐	☐	☐	☐	☐
CO12	Full of resources / in control	☐	☐	☐	☐	☐
PA13	Positive	☐	☐	☐	☐	☐
VI14	Strong	☐	☐	☐	☐	☐
TE15	Fighting	☐	☐	☐	☐	☐
PA16	Negative	☐	☐	☐	☐	☐
AN17	Full of worries	☐	☐	☐	☐	☐
CO18	Not ready to deal with the world	☐	☐	☐	☐	☐
VI19	Without energy	☐	☐	☐	☐	☐
CO20	Without resources / not in control	☐	☐	☐	☐	☐
VI21	Without strength	☐	☐	☐	☐	☐
DA22	Not depressed	☐	☐	☐	☐	☐
DA23	Full of life	☐	☐	☐	☐	☐
AN24	Not anxious	☐	☐	☐	☐	☐

Note. The HSI-24 has six dimensions: TE = Tension (e.g., fighting – indulgent), PA = Positive Affect (e.g., positive – negative), AN = Anxiety (e.g., anxious – not anxious), DA = Depressed Affect (e.g., depressed – not depressed), CO = Coping (e.g., full of resources – without resources*), and VI = Vitality (e.g., full of energy – without energy). *the term “resources” did not work well in the German version, most persons needed an explanation (as an alternative we put “in control - not in control” here for future uses); the questionnaire dimensions were developed from theory and empirical findings in dance movement therapy (Goodill, 2006); factor analyses show an underling general factor (well-being or positive vs. negative affect); internal consistencies have yielded *Cronbach’s Alphas* between .68 and .97.

Appendix C: Scale for Therapeutic Factors of Arts Therapies in PD (Secondary Outcome Measure; Koch & Mergheim, 2015)

Dear participant,

Welcome! Please fill in the questionnaire as spontaneously and completely as possible and mark each row with a cross, with “1” signifying „not at all“ and “10” signifying „very much“.

When you think back to the last hour…

1. How happy have you been?

   1 2 3 4 5 6 7 8 9 10

2. How much beauty did you experience?

   1 2 3 4 5 6 7 8 9 10

3. How much pleasure/joy did you experience?

   1 2 3 4 5 6 7 8 9 10

4. How well could you express your feelings?

   1 2 3 4 5 6 7 8 9 10

5. How fluently could you verbally express yourself?

   1 2 3 4 5 6 7 8 9 10

6. How fluent were your movements?

   1 2 3 4 5 6 7 8 9 10

7. How close was your experienced connection between body and mind?

   1 2 3 4 5 6 7 8 9 10

8. How strongly did you experience the unison with your partner?

   1 2 3 4 5 6 7 8 9 10
